# Arfid Genes and Environment (ARFID-GEN): Study Protocol

**DOI:** 10.21203/rs.3.rs-3186174/v1

**Published:** 2023-08-31

**Authors:** Cynthia M. Bulik, Nadia Micali, Casey M MacDermod, Baiyu Qi, Melissa A Munn-Chernoff, Laura M Thornton, Jennifer White, Lisa Dinkler, Emily M. Pisetsky, Jessica Johnson, Katelin R Devine, Shelby N Ortiz, Ava E Silverman, Natasha Berthold, Alexis Dumain, Jerry Guintivano, Matthew Halvorsen, J James

**Affiliations:** University of North Carolina at Chapel Hill; Mental Health Services of the Capital Region of Denmark; University of North Carolina at Chapel Hill; University of North Carolina at Chapel Hill; University of North Carolina at Chapel Hill; University of North Carolina at Chapel Hill; University of North Carolina at Chapel Hill; Karolinska Institutet; University of North Carolina at Chapel Hill; University of North Carolina at Chapel Hill; University of North Carolina at Chapel Hill; University of North Carolina at Chapel Hill; University of North Carolina at Chapel Hill; University of North Carolina at Chapel Hill; University of North Carolina at Chapel Hill; University of North Carolina at Chapel Hill; University of North Carolina at Chapel Hill; University of North Carolina at Chapel Hill

**Keywords:** avoidant restrictive food intake disorder, picky eating, selective eating, eating disorders, genome-wide association, psychiatric genetics, psychiatric genomics consortium, social media

## Abstract

**Background:**

The Avoidant Restrictive Food Intake Disorder Genes and Environment (ARFID-GEN) study is a study of genetic and environmental factors that contribute to risk for developing ARFID in children and adults.

**Methods:**

A total of 3,000 children and adults with ARFID from the United States will be included. Parents/guardians and their children with ARFID (ages 7 to 17) and adults with ARFID (ages 18+) will complete comprehensive online consent, parent verification of child assent (when applicable), and phenotyping. Enrolled participants with ARFID will submit a saliva sample for genotyping. A genome-wide association study of ARFID will be conducted.

**Discussion:**

ARFID-GEN, a large-scale genetic study of ARFID, is designed to rapidly advance the study of the genetics of eating disorders. We will explicate the genetic architecture of ARFID relative to other eating disorders and to other psychiatric, neurodevelopmental, and metabolic disorders and traits. Our goal is for ARFID to deliver “actionable” findings that can be transformed into clinically meaningful insights.

**Trial registration:**

ARFID-GEN is a registered clinical trial: clinicaltrials.gov NCT05605067

## Background

We describe the *Avoidant Restrictive Food Intake Disorder Genes and Environment (ARFID-GEN)* study, which is designed to expand the discovery of genetic and environmental contributions to ARFID risk. ARFID-GEN builds on previous and ongoing genome-wide association studies (GWAS) by the Eating Disorders Working Group of the Psychiatric Genomics Consortium (PGC-ED) as part of a global effort to fully characterize the genetic architecture of all eating disorders (EDs) and explore their relation to each other and to other psychiatric, neurodevelopmental, and metabolic/anthropometric traits.

ARFID-GEN will ascertain, phenotype, and genotype a large sample of children and adults with ARFID. We will apply advanced analytic strategies to test and refine an etiological model of ARFID, explicate heterogeneity, and simultaneously document environmental risk factors for ARFID.

ARFID is associated with high personal and family emotional and financial cost. ARFID, present in 2–5% of the population (1), is marked by the avoidance and/or restriction of food intake resulting in significant weight loss or nutritional deficiency, dependence on feeding supplements, and/or interference with psychosocial functioning. Unlike other EDs, food restriction is not driven by weight and shape concerns, and ARFID may be equally common in males and females (1). The Diagnostic and Statistical Manual of Mental Health Disorders 5th edition (DSM-5) (2) describes three predominant ARFID presentations that likely overlap (3): (1) sensory sensitivity (i.e., rejection of food based on sensory qualities such as texture), (2) phobic avoidance of food (i.e., concern about aversive consequences of eating, such as fear of choking), and (3) low interest/appetite. Psychiatric, neurodevelopmental, and somatic medical comorbidities are common, with ~ 50% of ARFID cases having a co-occurring diagnosis (4).

Using existing Swedish twin data, we demonstrated that an ARFID phenotype is highly heritable (twin heritability estimate [h^2^_twin_] = .79; CI:.71, .86), with the remaining variance attributable to nonshared environmental factors (5). This places ARFID amongst the most heritable psychiatric disorders and on par or higher than anorexia nervosa (AN) (.50-.60) (6), bulimia nervosa (BN) (.50-.60) (6), and binge-eating disorder (BED) (.39-.57) (7, 8). These results support a GWAS for ARFID.

The three key dimensions of ARFID identified are viewed as symptom clusters rather than distinct presentations. The sensory dimension encompasses rejection of food based on sensory qualities (e.g., taste, smell, texture, temperature, appearance) and is the most common reason for referral (9). When phobic avoidance is the dominant presentation, conditioned aversion may play a role (10, 11). A genetic predisposition to anxiety may increase the risk of children developing ARFID after an aversive feeding experience like gagging or witnessing someone choke or vomit (12). The low interest/appetite dimension likely captures symptoms previously described as infantile anorexia and food avoidance emotional disorder (13).

## Methods

### Specific Aims.

#### Aim 1a. Ascertainment of 3,000 ARFID cases.

Leveraging the existing Eating Disorders Genetics Initiative (EDGI)(14) infrastructure at the University of North Carolina at Chapel Hill (UNC), we will ascertain 3,000 children and adults with ARFID. Appropriate controls will be sourced from archived, genotyped repositories such as the National Institute of Mental Health (NIMH) Genomics Repository, database of Genotypes and Phenotypes (dbGaP). We will conduct efficient online phenotyping of children and adults with ARFID including environmental exposures and at-home saliva sampling for deoxyribose nucleic acid (DNA). We will genotype new samples using contemporary methodology.

#### Aim 1b. Validation substudy.

We will interview parents/guardians of 25 children with ARFID and 25 adults with ARFID with the Pica, ARFID and Rumination Disorder Interview (PARDI) (15) to further validate our online ARFID diagnostic battery.

#### Aim 2: Within-disorder ARFID GWAS.

We will conduct comprehensive phenotypic and genomic analyses: single nucleotide polymorphism (SNP)-based heritability, GWAS (imputable to minor allele frequency [MAF] ≥ 0.005), genetic correlations (r_g_s), polygenic risk scores (PRS), standard post-GWAS analyses of the non-mutually exclusive ARFID presentations, and rare copy number variants (CNVs) and CNV burden. Hypotheses: We will identify genome-wide significant loci for ARFID, informative r_g_s, implicated CNVs, and environmental precipitants.

#### Aim 3. Genetic relation of ARFID to other eating disorders.

We will test if ARFID shares a core set of genetic factors with other EDs yet is differentiated by disorder-specific genetic factors. We will conduct a set of cross-disorder genomic analyses to map genetic interrelations between ARFID and other EDs including: (a) cross-disorder GWAS meta-analysis to identify loci with pleiotropic effects, (b) if indicated, calculate r_g_s and conduct Mendelian Randomization (MR), multi-trait conditional and joint analysis (mtCOJO) (16), disorder-specific SNP associations, Multi-PRS (17), and, (c) genomic structural equation modelling (GSEM) (18) to examine genome-wide architecture of ARFID relative to other EDs. Based on our preliminary data, we predict that ARFID will show the strongest genetic association with AN.

#### Aim 4. Genetic relation of ARFID with psychiatric, metabolic/anthropometric, neurodevelopmental, and other relevant phenotypes.

To test a conceptualization that ARFID has specific genetic associations with psychiatric, metabolic/anthropometric, and neurodevelopmental phenotypes we will apply: (a) approaches as in Aim 3b, and (b) GSEM to examine genetic, psychiatric, neurodevelopmental, and metabolic/anthropometric factors associated with ARFID. We predict that high sensory sensitivity will be related to autism spectrum disorder (ASD), high levels of phobic avoidance to anxiety and obsessive-compulsive disorder (OCD), and high levels of low interest/appetite to AN. *Deliverables:* (a) dissection of converging and diverging relations among ARFID and other traits informing and refining its etiology; (b) genetic assessment of ARFID’s relation to other phenotypes, informing nosology.

### Participants.

#### Objective.

We will engage the infrastructure utilized in EDGI (14) to ascertain 3,000 children and adults with ARFID. Controls will be ascertained from data repositories from other genomic studies.

##### Case definition (ARFID).

Inclusion criteria. Individuals ages 7 + who meet DSM-5 criteria for ARFID. An age 7 + cut-off allows adequate phenotyping with a range of valid instruments available for relevant constructs for this age and older. We anticipate most cases will be pediatric as ARFID typically onsets in childhood. Following a brief online eligibility pre-screen, participants/parents/guardians will be screened for eligibility (for themselves or, in the case of parents/guardians, for their children) and for diagnostic purposes using the questionnaires listed in **Supplement** and Table 1. Exclusion criteria: current Eating Disorder Examination-Questionnaire (EDE-Q) global score greater than 4.0 or self-induced vomiting, laxative use, or more than four episodes of loss of control eating/binge eating in the past 28 days (DSM-5 ARFID diagnosis requires rule-out of other eating disorders). However, we will carefully monitor those screening eligible and ineligible for the study to evaluate this exclusion criterion, because adequate longitudinal data do not yet exist documenting the frequency with which ARFID may transition to other eating disorders over time.

### Recruitment.

We will use a multi-pronged recruitment approach including: outreach to ED clinicians and programs across the country, traditional media (press releases and newspaper announcements), and social media and other online platforms (websites, Facebook, Twitter, Instagram, and podcasts). This includes use of social media ads on Facebook and Instagram, which have proven successful for other similar studies (19). We enrolled a group of ARFID-GEN parent stakeholders who have consulted with us on study design prior to finalizing our methods.

### Procedure.

#### Self-report measures.

Table 1 presents the age-appropriate assessment instruments that are completed by parents/guardians of children with ARFID, children with ARFID (ages 7–17 with variable age-appropriate formats), and adults with ARFID (ages 18+). The battery includes validated instruments that capture: ARFID diagnosis and symptoms; other eating disorder diagnoses and symptoms; co-occurring psychiatric disorders and symptoms; general health and neurodevelopmental disorders; impairment; and environmental exposures. Complete information on all self-report questionnaires is available in the **Supplement.**

#### Parents/Guardians of children ages 7–17 and their children.

Parents/guardians of children with ARFID who are interested in participating in the study will visit the website (arfidgen.org) and select “Take Our Survey” with the child available. The first step requires the parent/guardian to answer prescreen questions and to consent to the study which includes answering questionnaires and having their child provide a saliva sample for DNA extraction. Parents also have the option to consent to be recontacted for future research. To determine eligibility of the child, parents/guardians then complete a parent report version of the Nine Item ARFID Screen (NIAS-PR) (20); a parent version of the ARFID self-report version of the Pica, ARFID, and Rumination Disorder Interview ARFID questionnaire (PARDI-AR-Q) (21); and Version 2.0 of the Parent Version of the Eating Disorder Examination-Questionnaire (PEDE-Qv2.0)(22), developed by KL Loeb based on the Eating Disorder Examination-Questionnaire Version 6 (EDE-Qv6) (23). If the child is eligible based on parent/guardian report, the parent/guardian provides contact information, the child provides assent to participate, and the parent/guardian acknowledges that the child provided verbal assent as well.

**Figure 1:**
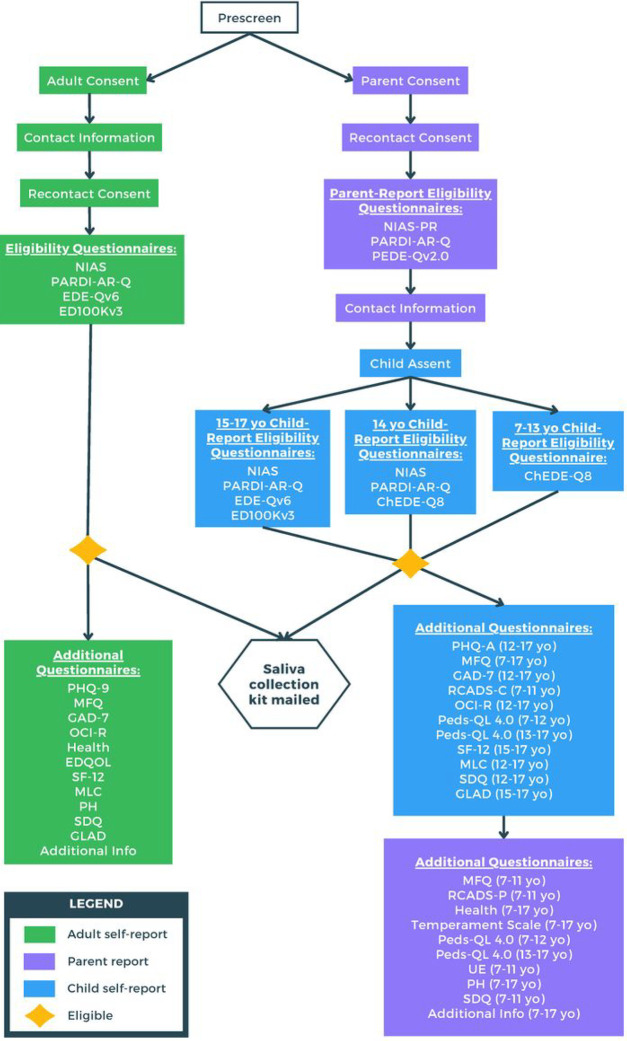


Children ages 7–13 then complete the ChEDE-Q8 (24), a child version of the 8-item short form of the Eating Disorders Examination-Questionnaire (25). Children age 14 complete the NIAS (20), PARDI-AR-Q (21), and ChEDE-Q8. Children ages 15–17 complete the NIAS, PARDI-AR-Q, EDE-Qv6, and the ED100Kv3 (26). Embedded algorithms determine if the child is eligible for the study. If they meet criteria, they are considered enrolled. All consents, assents, and study questionnaires are completed online using Research Electronic Data Capture (REDCap)(27). Table 1 contains the series of assessments and which version is used. If the child is determined eligible by parent/guardian- and self-report, they are asked to complete additional questionnaires and provide a saliva sample. A saliva sample collection kit for the child with return packaging is mailed to the parent/guardian with directions addressed to the parents/guardian to oversee the saliva collection from the child. Parents/guardians mail back the completed saliva sample. Once the kit is received by the study team and all questionnaires are finished, participation in the study is complete. For a flowchart of study procedures, see Fig. 1.

#### Adults (age 18+) with ARFID.

Adults visit the website (arfidgen.org) and click the “Take Our Survey” link. Participants complete a brief pre-screen, providing online informed consent for the entire study, have the option to consent to be recontacted for future studies, and provide contact information. Next, they complete the NIAS and the adult version of the PARDI-AR-Q to confirm the presence of ARFID, the EDE-Qv6 to rule out other current eating disorder symptoms, and the ED100Kv3. Surveys are presented in REDCap (27). Embedded algorithms determine if participants meet inclusion criteria (DSM-5 criteria for ARFID and no other current eating disorder symptoms that warrant exclusion). If they meet criteria, they are considered enrolled. Enrolled participants are asked to complete additional questionnaires (Table 1) and provide a saliva sample. A saliva collection kit is mailed to the participant’s home and the completed sample is returned. Participation in the study is complete once the kit is received by the study team and all questionnaires are finished. For a flowchart of study procedures, see Fig. 1.

#### Saliva sampling.

Saliva samples are collected with Isohelix saliva collection kits and returned to the Center for Psychiatric Genetics (CPG) Biorepository at UNC.

#### Gift cards.

Parents and adult participants are sent a gift card ($25) once all required questionnaires are complete and their spit kit is received by the study team.

#### DNA extraction and genotyping.

DNA extraction and GWAS genotyping are standard. We will use the most contemporary chip appropriate for diverse ancestry populations when genotyping occurs.

### Planned Data Analysis.

#### Aim 1a. Ascertainment of 3,000 ARFID cases.

We will conduct descriptive analyses to characterize the sample stratified by pediatric and adult cases. We will report demographics, symptom patterns, onset, course of illness, comorbid psychiatric conditions, neurodevelopmental characteristics, environmental exposures, and health-related quality of life in child (parent/guardian and child report) and adult ARFID cases.

#### Aim 1b. Validation study.

Diagnoses obtained with the PARDI interview will be compared with those obtained with the online ARFID-GEN battery. We will calculate positive predictive value (PPV) to confirm diagnostic properties of our ARFID-GEN battery. Data on weight, height, body mass index (BMI), and BMI percentile (for those < 18) will be verified during remote interviews.

#### Aim 2: Within disorder ARFID GWAS.

We will conduct a comprehensive set of genomic analyses including SNP-based heritability, GWAS, r_g_s, and PRS, and standard post-GWAS analyses of ARFID and the non-mutually exclusive ARFID presentations, and rare CNVs and CNV burden. The following sections briefly summarize the Aim 2 analyses; a more detailed description can be found in the **Supplement**.

##### a) PGC Ricopili pipeline supports rapid analysis.

As described in our previous PGC-ED publications (28), and the **Supplement**, we will use “Ricopili” software (29) for pre-imputation quality control, principal components analysis (PCA), imputation, and meta-analysis. Briefly, we will first follow standard methods to retain high quality SNPs and subjects. Next, we will perform imputation using the largest available resources (currently Haplotype Reference Consortium [HRC]) (30) updating to Trans-omics for Precision Medicine (TOPMed) (31) (N = 65K 30x whole genome sequencing [WGS]). Ancestry will be assessed using PCA for each subject, mapped relative to reference samples of known ancestry. Consistent with our intention to include non-European ancestries, we will use established Psychiatric Genomics Consortium (PGC) cross-ancestry analytical approaches.

##### b) Post-GWAS analyses.

The following outlines standard analytic strategy for post-GWAS analysis to maximize information yield and interpretability. The field and methodology evolve rapidly. Below represents what we would do today; however, novel proven methods may emerge before analyses are conducted. Greater detail can be found in the **Supplement**.

#### Analysis of chrX.

X chromosome (chrX) variants in the pseudo-autosomal regions will be handled separately. SNPs with MAF > 0.01 and INFO > 0.70 will be retained.

#### Females and Males.

We will conduct secondary GWAS analysis separately on females and males to determine similarity of the results to the primary combined GWAS.

#### Clumping.

GWAS results implicate genomic regions (“loci”). To define a locus, SNPs with *P*< 5×10^−8^ will be identified and “clumping” will be used to convert significant SNPs to regions.

#### Conditional and joint analysis.

Conditional and joint analysis will be conducted using genome-wide complex trait analysis-conditional and joint analysis (GCTA-COJO) (32). GCTA-COJO investigates every locus with a joint combination of independent markers via a genome-wide SNP selection procedure.

#### Functional genomic integration.

We now routinely use functional genomic results from CommonMind, PsychENCODE, and other efforts to understand GWAS results (33). Much of this is automated in the Functional Mapping and Annotation of Genome-Wide Association Studies platform (FUMA) (34). We also integrate brain single cell ribonucleic acid sequencing (RNA-seq) data to identify the cell types implied by the GWAS results. See the PGC major depressive disorder (MDD) paper for examples (35).

#### SNP-based heritability.

Linkage disequilibrium score regression (LDSC) will be used to estimate SNP-based heritabilities for ARFID and related presentations (36, 37).

#### Polygenic risk scores (PRS).

PRS aggregate risk alleles across the genome weighted by effect sizes. We will use both the classical p-value thresholding method and a summary statistics version of Bayesian multiple regression (SBayesR) (38).

#### ARFID presentation analysis.

ARFID presentations are overlapping. We will examine the association between ARFID PRS and: a) PARDI-AR-Q subscale scores (sensory sensitivity, phobic avoidance, and low interest/appetite) as continuous scores; and b) a variable categorizing individuals as having a predominant ARFID presentation (e.g., sensory vs. phobic vs. low interest/appetite) defined as highest subscale score.

#### Gene-wise analysis.

Multi-marker analysis of genomic annotation (MAGMA) (39) will be used to perform gene-wise tests of association with ARFID based on GWAS summary statistics. MAGMA generates gene-based p-values by combining SNP-based p-values within a gene while accounting for linkage disequilibrium (LD).

#### Partitioned heritability.

Partitioned heritability will be investigated using stratified LDSC (40), which estimates the per-SNP contribution to overall SNP-heritability (SNP-*h*^2^) across functional annotation categories.

#### Gene expression.

We will investigate whether ARFID heritability is enriched in tissue/cell type specifically expressed genes using publicly available gene expression data (e.g., the Genotype-Tissue Expression project [GTEx]).

#### Predicted tissue-specific gene expression.

We will predict differential gene expression using S-PrediXcan v1.0 (41) and genomic and transcriptomic reference data from the brain regions assayed in CommonMind, GTEx and other resources.

#### Pathway analyses.

We will evaluate whether genes associated with ARFID are enriched in specific pathways, tissues, or cell types. To do this, we will use FUMA to annotate SNPs, identify independent loci, perform pathway analysis, and integrate with a wide array of functional genomic data including gene expression, single cell gene expression, and all available brain epigenetic information.

##### c) Discover structural variation associated with ARFID.

The PGC CNV working group is actively optimizing CNV calling using global screening array (GSA) data and we will follow these developments closely. We will maximize comparability between cases and controls by applying rigorous quality control (QC) (42–44). Following QC, we will fit a series of linear models with CNV burden (at different size thresholds) as the dependent variable to investigate both disease status and experimental biases that can potentially confound CNV detection.

#### Aim 3. Genetic relation of ARFID to other eating disorders.

Greater detail on Aim 3 analyses can be found in the **Supplement**.

##### Analytic plan, a) Disorder-specific GWAS.

We will conduct disorder-specific GWAS for ARFID (and AN, BN, and BED as part of other projects) combining ARFID-GEN with EDGI and existing PGC-ED data using imputed variant dosages and an additive model. We anticipate aggregate sample sizes for the PGC-ED by ~ 2025 (when our collection would be complete) of: (Projected: ARFID = 3,000, AN = 50,000, BN = 20,000, BED = 20,000, ARFID-GEN controls [sourced from existing data repositories] = 42,439, PGC-ED controls = 543,967). GWAS meta-analysis will be conducted with ARFID-GEN and any other ARFID samples that may be introduced to the PGC-ED by time of analysis.

##### Combined eating disorder GWAS.

Combining ARFID-GEN with PGC-ED GWAS of AN, BN, and BED (which will be available by the time our data are analysis-ready), conduct GWAS meta-analysis of all EDs and of component behaviors that cross-cut disorders (e.g., binge eating, restriction), increasing power to identify genetic risk factors that are common across the four disorders.

##### b) Genetic correlations.

Common variant-based genetic correlations (SNP-*r*_g_) measure the extent to which two traits or disorders share common genetic variation. We will calculate SNP-*r*_g_ for ARFID, and selected traits using GWAS summary statistics via an analytical extension of LDSC (36, 37), as well as explore genome-based restricted maximum likelihood (GREML), which was recently shown to have higher accuracy than LDSC (45). Risk factors classically considered as environmental, such as exercise for AN, are now known to be complex traits underpinned by genetic and non-genetic factors. Large community cohorts like United Kingdom (UK) Biobank, generate GWAS summary statistics for a plethora of phenotypes (e.g., diet, medication, blood metabolites). It is very fast to estimate r_g_ from GWAS summary statistics, and this screening process has the potential to identify unknown associations which will guide the hypotheses of down-stream analyses.

##### Generalized summary data-based Mendelian randomization (GSMR).

Guided by the *r*_g_ estimated above and our hypotheses, we will perform bidirectional MR analyses to investigate causal relationships between correlated traits and ARFID. For example, it is reasonable to test hypotheses of causality using anxiety as an exposure for ARFID. Significant MR results must be reviewed with caution with respect to strong conclusions about causality as unmeasured confounders may exist. Nonetheless, these analyses are potentially exceptionally informative. MR analyses take SNPs that are genome-wide significant for one trait (the exposure) and test the correlation in effect sizes in a second trait (the outcome). Under pleiotropy, there is an expectation that the mean effect size in the outcome trait is different from zero, but under causality a directional relationship in effect sizes is expected. Different versions of MR analysis are highly related; at the time of analysis, we will implement best practices for MR.

##### Multi-trait-based conditional and joint analysis.

Results from *a)* and *b)* will inform these analyses. For example, we expect to detect genetic correlations of BMI with EDs, and MR analyses will aid interpretation of correlation by causality. We will conduct conditional GWAS analyses to determine if the detected SNP associations for EDs can be explained through their relationship with correlated traits. We will perform a multi-trait-based conditional and joint analysis (GCTA-mtCOJO) (16) using an extension of GCTA (46). This method uses summary-level data to perform conditional analyses. Based on our previous work, we expect to condition the results of our ARFID GWAS on the best available GWAS results for relevant traits including intelligence quotient (IQ), education years, type 2 diabetes, high density lipid cholesterol, BMI, schizophrenia (SCZ), MDD, ASD, OCD, and neuroticism. Comparing these results to those generated from unconditional GWAS results (i.e., from *a* above) provides insights into the forces shaping the shared genetic relationships between disorders.

##### Disorder-specific SNP associations.

A key question in ED research is to understand the differences as well as the similarities among the EDs. This question is very similar to the one posed by PGC colleagues interested in understanding the similarity/differences between SCZ and bipolar disorder (BIP) (47). Guided by their analyses, we will conduct case vs. case (e.g., ARFID-AN) GWAS. This approach is powerful if case samples can be grouped (e.g., if genotyped together so that technical confounding factors are not present), as sampling errors associated with control allele frequency estimates are avoided.

##### Multi-polygenic score (MPS).

To gain insight into factors underlying ED heterogeneity, we will use the largest available GWAS summary statistics for psychiatric and somatic disorders/traits and combine derived PRS into MPS to predict target outcomes (ARFID, AN, BN, or BED diagnosis), and then more granular phenotypes (e.g., age of onset, severity, low weight). Combining *ARFID-GEN* with EDGI and existing AN, BN, and BED cohorts in the PGC-ED samples should yield a sample size of ~ 95,000 ED cases and > 500,000 controls. We will increase power by using MPS to combine the predictive power of several PRS in one regression model. Training data will combine the best available GWAS summary statistics on psychiatric, metabolic, BMI, anthropometric, personality, physical activity, and educational phenotypes. These PRS will be used as genetic predictors in models of ARFID, AN, BN, BED, and more granular phenotypes. This approach is ideal for our overarching intentions of predicting outcomes rather than discovering their etiology (i.e., using, not finding genes). MPS is valuable when trait prediction is a priority.

##### c) GSEM.

We will use lifetime ARFID, AN, BN, and BED GWAS summary statistics. We will employ GSEM (18) to identify genetic factors for ARFID and associated EDs. GSEM is a multivariate method for analyzing the joint genetic architecture of complex traits. By modeling covariance structure, GSEM synthesizes genetic correlations and SNP heritabilities inferred from GWAS summary statistics of individual traits from samples with varying and unknown degrees of overlap. GSEM analyses will include several steps, including a factor analysis of correlated traits, estimating SNP effects and computation of factor-level PRS.

#### Aim 4. Genetic relation of ARFID with psychiatric, metabolic/anthropometric, neurodevelopmental, and other relevant phenotypes.

Analyses will parallel Aim 3 only with an outward focus on traits other than EDs. For r_g_s, we will follow methods applied in the Anorexia Nervosa Genetics Initiative-PGC-ED (ANGI-PGC-ED) Freeze 2 analysis (28), adding additional traits as GWAS summary statistics become available. Only GWAS summary statistics are needed for GSEM, many of which are publicly available. Phenotypes of interest are not directly evaluated in the target sample. Related traits for GSEM may include: lifetime anxiety disorder (ANX), neuroticism, BMI, fat mass, and fat-free mass (available in the UK Biobank); ASD, attention-deficit/hyperactivity disorder (ADHD), post-traumatic stress disorder (PTSD), MDD, and OCD GWAS summary statistics (available from the PGC).

## Discussion

In addition to the science, we will create rich data and sample resources for the pursuit of related research questions. Our analytic aims are dense, but results will inform follow-on research questions such as: 1) How do genetic and environmental exposures act and co-act to influence risk for ARFID? 2) If detected, how will carriers of CNVs differ on PRS and environmental exposures? 3) Can we identify genetic factors that influence course of illness (e.g., predict who is at risk for developing persistent vs. transient ARFID)? 4) Can we answer precision-medicine questions regarding identification of optimal interventions informed by genotype and environmental exposures? Given the paucity of effective interventions for ARFID (and other EDs) (48), ultimately, we hope our work will yield information on critical biological pathways that may point toward drug discovery or repurposing that could aid in reversing the tenacity and lethality of these illnesses.

## Figures and Tables

**Table 1:** ARFID-GEN Assessment Battery

Domain	Assessment	Version	Parent or self-report	Age
ARFID symptoms and diagnosis				
ARFID symptoms	Nine Item ARFID Screen (NIAS)	NIAS (20)	Self	14+
		NIAS-Parent Report (NIAS-PR) (20, 49)	Parent	7–17
ARFID diagnosis	Pica, ARFID, and Rumination Disorder Interview-ARFID-Questionnaire (PARDI-AR-Q)	PARDI-AR-Q (21)	Self	14+
		PARDI-AR-Q parent (21)	Parent	7–17
**Other Eating disorder pathology**				
Lifetime eating disorder diagnoses	ED100K Version 3 (ED100Kv3)	ED100Kv3 (26)	Self	15+
Current eating disorder symptoms (last 28 days)	Eating Disorder Examination-Questionnaire (EDE-Q)	EDE-Qv6 (23)	Self	15+
		Child Version of the Eight Item EDE-Q (ChEDE-Q8) (24)	Self	7–14
		Version 2.0 of the Parent Version of the EDE-Qv6 (PEDE-Qv2.0) (22)	Parent	7–17
**Depression, anxiety, and neurodevelopment**				
Current depressive symptoms	Patient Health Questionnaire (PHQ)	PHQ-9 (50)	Self	18+
		PHQ-Adolescents (PHQ-A)* (51)	Self	12–17
Current depressive symptoms (last two weeks)	Short Mood and Feelings Questionnaire (MFQ) (52)	MFQ (52, 53)	Self	18+
		MFQ child version (52, 53)	Self	7–17
		MFQ parent version (52, 53)	Parent	7–11
Current anxiety symptoms	Generalized Anxiety Disorder-7 (GAD-7)	GAD-7 (54)	Self	12+
Major depression and anxiety disorders	Revised Child Anxiety and Depression Scale (RCADS)	RCADS-Child Version (RCADS-C)^†^(55) (56)	Self	7–11
		RCADS-Parent Version (RCADS-P)^†^ (57, 58)	Parent	7–11
Obsessive-compulsive symptoms	Obsessive-Compulsive Inventory-Revised (OCI-R)	OCI-R (59)	Self	12+
Intellectual/developmental disability	Three intellectual/developmental questions (developed for this study)	*N/A*	Parent	7–17
Temperament	One Item Temperament Scale	Temperament (60)	Parent	7–17
Detailed assessment of major depressive disorder and generalized anxiety disorder	Items from the mood and anxiety questionnaire for the Genetic Links to Anxiety and Depression Study (GLAD) (61)	GLAD mood & anxiety items (GLAD)‡	Self	15+
**Impairment**				
Eating disorder-specific health related quality of life	Eating Disorders-Quality of Life (EDQOL)	EDQOL (62, 63)	Self	18+
General health-related quality of life	Short Form Health Survey-12 (SF-12)	SF-12 (64)	Self	15+
Health-related quality of life	Pediatric Quality of Life Inventory 4.0 (Peds-QL 4.0)	Peds-QL 4.0 for children (65)	Self	7–12
		Peds-QL 4.0 for adolescents (65)	Self	13–17
		Peds-QL 4.0 for parents of children (65)	Parent	7–12
		Peds-QL 4.0 for parents of adolescents (65)	Parent	13–17
General health	General health questions (developed for this study)	Adult version	Self	18+
		Parent version	Parent	7–17
**Environmental exposures**				
Negative life events	Avon Longitudinal Study of Parents and Children (ALSPAC) life events checklists (66)	Major Life Changes (MLC)‡ (67)	Self	12+
		Upsetting Events (UE)‡ (67)	Parent	7–11
Mother's pregnancy history	Pregnancy History Questionnaire (PH)(68)	PH	Self	18+
		PH parent version	Parent	7–17
Psychological health	Strengths and Difficulties Questionnaire (SDQ) (69)	SDQ adult single-sided version (70)	Self	18+
		SDQ child single-sided version (70)	Self	12–17
		SDQ parent single-sided version (70)	Parent	7–11
**Other**				
Experiences with ARFID	Free response question for other comments on experiences with ARFID (developed for this study)	Adult version	Self	18+
		Parent version	Parent	7–17

* Item 9: "Thoughts that you would be better off dead, or of hurting yourself in some way?" was not included

† Items about major depressive disorder (MDD) were not included

‡ Modified for this study

## Data Availability

Our liberal data and analysis sharing principles will make phenotypic and genotype data and scripts widely available for access by other scientists to maximize utility of our investigation. The datasets generated and/or analyzed will be available in the National Data Archive (https://nda.nih.gov/). Genomic data access is made possible by the Psychiatric Genomics Consortium Data Access Committee. Step-by-step procedures are thoroughly described here (https://www.med.unc.edu/pgc/shared-methods/open-source-philosophy/). DNA samples will be available from the NIMH Repository and Genomics Resource (https://www.nimhgenetics.org/order-biosamples/how-to-order-biosamples).

## Abbreviations

ADHD: Attention-deficit/hyperactivity disorder
ALSPAC: Avon Longitudinal Study of Parents and Children
AN: Anorexia nervosa
ANGI: Anorexia Nervosa Genetics Initiative
ANX: Anxiety disorder
ARFID: Avoidant/restrictive food intake disorder
ARFID-GEN: Avoidant Restrictive Food Intake Disorder: Genes and Environment Study
ASD: Autism spectrum disorder
BED: Binge-eating disorder
BIP: Bipolar disorder
BMI: Body mass index
BN: Bulimia nervosa
ChEDE-Q8: Child version of the 8-item short form of the Eating Disorders Examination-Questionnaire
chrX: X chromosome
CNV: Copy number variants
dbGaP: Data base of Genotypes and Phenotypes
DNA: Deoxyribonucleic acid
DSM-5: Diagnostic and Statistical Manual of Mental Disorders 5^th^ Edition
ED: Eating disorder
ED100Kv3: ED100K Version 3
EDE-Q: Eating Disorders Examination-Questionnaire
EDE-Qv6: Eating Disorders Examination-Questionnaire Version 6
EDGI: Eating Disorders Genetics Initiative
EDQOL: Eating Disorders Quality of Life
FUMA: Functional Mapping and Annotation of Genome-Wide Association Studies platform
GAD-7: Generalized Anxiety Disorder-7
GCTA: Genome-wide complex trait analysis
GTCA-COJO: Genome-wide complex trait analysis-conditional and joint analysis
GLAD: Genetic Links to Anxiety and Depression Study
GREML: Genome-based restricted maximum likelihood
GSA: Global screening array
GSEM: Genomic structural equation modelling
GSMR: Generalized summary data-based Mendelian randomization
GTEx: Genotype-Tissue Expression project
GWAS: Genome-wide association study
h^2^: Heritability estimate
HRC: Haplotype Reference Consortium
IQ: Intelligence quotient
LD: Linkage disequilibrium
LDSC: Linkage disequilibrium score regression
MAF: Minor allele frequency
MAGMA: Multi-marker analysis of genomic annotation
MDD: Major depressive disorder
MFQ: Short Mood and Feelings Questionnaire
MLC: ALSPAC Major Life Changes
MPS: Multi-polygenic score
MR: Mendelian randomization
mtCOJO: Multi trait conditional and joint analysis
NIAS: Nine Item ARFID Screen
NIAS-PR: Nine Item ARFID Screen-Parent Report
NIMH: National Institute of Mental Health
OCD: Obsessive compulsive disorder
OCI-R: Obsessive Compulsive Inventory-Revised
PARDI: Pica, ARFID, and Rumination Disorder Interview
PARDI-AR-Q: Pica, ARFID, and Rumination Disorder Interview-ARFID-Questionnaire
PCA: Principal components analysis
PEDE-Qv2.0: Version 2.0 of the Parent Version of the Eating Disorders Examination-Questionnaire Version 6
Peds-QL 4.0: Pediatric Quality of Life Inventory 4.0
PGC: Psychiatric Genomics Consortium
PGC-ED: Eating Disorders Working Group of the Psychiatric Genomics Consortium
PH: Pregnancy History Questionnaire
PHQ: Patient Health Questionnaire
PHQ-9: Patient Health Questonnaire-9
PHQ-A: Patient Health Questionnaire-Adolescents
PPV: Positive predictive value
PRS: Polygenic risk scores
P_*T*_: p-value thresholds
PTSD: Post-traumatic stress disorder
QC: Quality control
RCADS: Revised Child Anxiety and Depression Scale
RCADS-C: Revised Child Anxiety and Depression Scale-Child Version
RCADS-P: Revised Child Anxiety and Depression Scale-Parent Version
REDCap: Research Electronic Data Capture
r_g_: Genetic correlation
RNA-seq: Ribonucleic acid sequencing
SBayesR: A summary statistics version of Bayesian multiple regression
SCZ: Schizophrenia
SDQ: Strengths and Difficulties Questionnaire
SF-12: Short Form Health Survey-12
SNP: Single nucleotide polymorphism
SNP-*r*_g_: Common variant based genetic correlations
TOPMed: Trans-omics for Precision Medicine
UE: ALSPAC Upsetting Events
UK: United Kingdom
UNC: University of North Carolina
WGS: Whole genome sequencing
